# Composition and Functional Effect of Mango (*Mangifera indica L*.) Almond Flours on Wheat Dough Rheology

**DOI:** 10.1155/2022/5899749

**Published:** 2022-12-30

**Authors:** Gaston Zomegni, Clement Saidou, Pierre Désiré Mbougueng, Robert Ndjouenkeu

**Affiliations:** ^1^Department of Textile and Leather Engineering, National Advanced School of Engineering, University of Maroua, P. O. Box 46 Maroua, Cameroon; ^2^Department of Basic Sciences, University Institute of Technology, The University of Ngaoundéré, P. O. Box 455 Ngaoundéré, Cameroon; ^3^Department of Process Engineering, National School of Agro-Industrial Sciences, The University of Ngaoundéré, P. O. Box 455 Ngaoundéré, Cameroon; ^4^Department of Food Science and Nutrition, National School of Agro-Industrial Sciences, University of Ngaoundéré, P. O. Box 455 Ngaoundéré, Cameroon

## Abstract

Composition and technofunctional properties of the almond flours of 12 mango varieties from savannah zones of Cameroon (Central Africa) are assessed in order to highlight their potential use in breadmaking, through their effect on dough rheology. The compositions of almonds display starch as the major constituent (60–65%) with more than 80% of amylopectin and a significant presence of phenolic compounds (1–7%), lipids (7–13%), and proteins (4–7%), depending on mango varieties, with local varieties showing the highest polyphenol and lipid contents. Almond flours are characterized by high WAC (water absorption capacity) and OAC (oil absorption capacity) and pasting properties influenced by starch lipid and starch protein complexes. In wheat-mango almond composite flours, the pasting properties are mango variety and almond flour treatment (native or delipidated) dependent, with a substitution threshold effect variable from one variety to another. Alveographic profiles of the composite flours result in dough characterized by high tenacity (P) but low swelling index (G) and baking strength (W). These effects are intensified with increasing substitution of wheat. However, up to 10% wheat substitution, the composite flours seem acceptable in breadmaking, with 9 mango varieties hypothetically convenient for the reduction of bread staling.

## 1. Introduction

Mango (*Mangifera indica L*.), with an estimated world production of more than 39 million tons, is the fifth fruit production after citrus fruits, grapes, bananas, and apples. The fleshy and juicy pulp of the fruit is eaten directly or processed into juice, nectar, jam, or dried. The kernel and the skin are generally discarded as waste, often causing environmental nuisance during the production season. However, the skin and particularly the almond of mango kernels, with regards to their composition and properties, display functional potential as food ingredients, antioxidant, antimicrobial, and cosmetics, carried by high content in polyphenols and lipids [1–4]. Moreover, the functionality of the above components has been positively tested in baking, particularly in the reduction of pizza and cake stale [5, 6]. In fact, many research findings have evidenced phenolic compound-starch and lipid-starch interactions in baking process, with induced effects on dough physicochemical and rheological properties (mixing time, mixing tolerance, dough strength, and viscoelasticity), bread-quality attributes (volume, texture, and sensory characteristics), and extended shelf-life of baking products [7–9]. Mango almond appears thus as a potential natural ingredient to fulfil the above properties in bread making.

In Cameroon, mango production is around 5 million tons, driven by a high varietal diversity which may result in functional diversity. This observation suggests the interest of a physicochemical and functional characterization of mango almonds produced locally, particularly their effect on dough behavior. Such a characterization is all the more relevant, since the almonds of African mango varieties have so far received low scientific and technological interest. This paper therefore is aimed at assessing the chemical composition and technofunctional properties of almonds from the different mango varieties in the savannah zone of Cameroon, one of the main production areas of this fruit in the country.

## 2. Material and Methods

### 2.1. Collection and Preparation of Samples

Twelve varieties of mangoes, among the most cultivated and consumed in the savannah zone of Northern Cameroon, were harvested at physiological maturity, peeled, and pitted, and the kernels shelled manually using stainless steel knives. The two cotyledons of the kernel almonds were separated and dried (40°C ± 1°C, ≈72 hours) in an electric forced-convection dryer, to a residual water content less than 10%. The dried almonds were then ground to a particle size of 200 *μ*m, using a Culatti hammer mill (Janke and Kunkel), and the resulting flour splits into two batches: one batch left as native mango almond flour and the second batch delipidated by extraction of oil with hexane using Soxhlet. Starch was extracted from the delipidated flour with SDS using the method of Mahmood et al. [10] for further characterization regarding a possible starch-lipid interaction.

### 2.2. Chemical Analyses of Mango Almond Flours

The water and ash contents of the mango almond flours were determined, respectively, by drying in an oven at 105°C for 24 hours and incinerating in a muffle oven (PROLABO no. 54786) at 550°C, until obtaining decarbonated white ash. Lipid content was determined by extraction with 95% hexane. The fatty acid composition of the oil was determined by gas chromatography under the following conditions: methylation with diethyl ether and tetramethylammonium, injection volume: 0.1 *μ*L, elution using BPX 70 column (30 m × 0.25 mm, Supelco, France) with temperature gradient of 120°C-250°C, 8.6°C/min, hydrogen as carrier gas, flame ionization detection at 280°C, and quantification using a calibration curve of fatty acid standards.

The protein content (N x 6.25) was determined by the Kjeldahl method.

Soluble sugars were extracted with 80% ethanol and determined using the DNS (3.5 dinitrosalicylic acid) colorimetric method [11].

The starch content was determined by enzymatic method [12], using *α*-amylase (444 units/mg solid; 570 units/mg protein, USA) and amyl glucosidase (≥50 units/mg protein, Japan) from Bacillus, for the hydrolysis of the polysaccharide into D-glucose and DNS for the determination of sugar released [11], which value was converted into starch using 0.9 as conversion factor [13]. The amylose content of starch was determined using the method of Jarvis and Walker [14], based on the formation of I_2_-Amylose complex displaying a strong blue color which intensity is measured at 720 nm. The amylose content of the sample was derived from a standard curve of potato amylose (N°. A-9262, USA) treated in the same conditions as the assays.

The crude fiber content was determined using the method of Wolff [15].

Total phenolic compounds were extracted with acetone (70%) and determined using the Folin Ciocalteu reagent with spectrophotometric measurement at 725 nm and a calibration curve with tannic acid as standard [16]. The tannin content was determined using the same procedure after treating the raw extract with polyvinylpolypyrrolidone (PVPP) (K30, Belgium) and subtracting the value obtained from that of the total phenolic compounds.

An assay to evaluate the possible presence of amylase in mango almond flours was performed by mixing an aqueous flour extract with a starch suspension and following the possible hydrolysis of the polysaccharide over time [17]. The aqueous flour extract was prepared in a 2 mM CaCl_2_ solution, in a pH 5.5 buffer, at 40°C, followed by centrifugation. The mixture was incubated at 60°C, and the optical density, representative of the residual starch content, recorded every 15 seconds for 3 minutes.

### 2.3. Functional and Technological Properties of Flours

Water absorption capacity (WAC) and oil absorption capacity (OAC) of almond flours were determined following the method described by Beuchat [18], while emulsion activity (EA) and emulsion stability (ES) of flours were determined using the procedure described by Yasumatsu et al. [19].

The evaluation of technological properties focused on the rheological behavior of wheat/almond flour mixtures according to ratios: 100/0, 95/5, 90/10, 85/15, and 80/20. The tests were performed with native and delipidated almond flours, as well as with starch extracted from mango almond flours and purified with sodium dodecyl sulfate (SDS) according to the method described by Mahmood et al. [10]. Rheological measurements performed on all flours included the following:

- Their hydrothermal behavior in dispersed solution, using the Rapid-Visco-Analyzer (RVA) (Newport Scientific, RVA-TecMaster, Perten). The flour suspension, previously homogenized by stirring at 960 rpm for 10s, was treated, still under stirring (160 rpm), according to the following diagram: (i) hold for 1 min at the initial temperature (50°C), (ii) temperature rise from 50°C to 95°C at a speed of 12°C/min, i.e., in 3.75 min, (iii) maintain at 95°C for 5 min, (iv) cooling from 95°C to 50°C at 12°C/min in 2.25 min, and (v) maintain at 50°C for 1 min
- Alveographic behavior (Chopin Alveograph, Tripette & Renaud, Villeneuve La Garenne, France) of their pastes according to the AACC standard method [20].

### 2.4. Statistical Analyses

All measurements were performed in triplicate, and the results expressed as mean ± standard deviation. The analysis of variance (ANOVA) of the means made it possible to compare the influence of the variation factors (mango variety, almond flour treatment (native or delipidated), and level of wheat substitution) on the responses measured, and Duncan test was used to rank significantly different means (*p* = 0.05).

## 3. Results and Discussion

### 3.1. Chemical Composition of Almonds from the Different Mango Varieties

The chemical composition of almonds of the different mango varieties (Table 1) shows a similarity with almonds of other African mango varieties [2, 21, 22]. This similarity is probably due to the fact that the same improved varieties of mango have been introduced in various African countries, though with possible space variability of names. Beyond the similarity of composition, the Cameroonian mango almond varieties seem to be distinguished by higher contents of soluble sugars (3-7% against less than 3% in the majority of African varieties) which may be justified, either by possible pedologic variation among countries or by the level of mango maturity at which experiments were undergone in the different spaces. The local varieties (Local Maroua and Local Ngaoundéré) present in our samples differ from the improved varieties by a relatively high lipid, amylose, and polyphenol contents.

Globally, the composition of mango almonds displays two characteristics that carry functional and technological potentials: (i) the significant presence of phenolic compounds confirms their proven application as a source of bioactive compounds, in particular through their antioxidant activity [1, 2] and paves the way for other potential applications, especially in breadmaking, where the addition of phenolic compounds to bread dough has shown antistale potential [8] as well as a significant effect on dough rheology [7]; (ii) starch is the major constituent (59-65%) for the different varieties, suggesting that this polysaccharide could carry physicochemical and functional properties and justifying the potential for using mango almond flour in breadmaking. This hypothetical assumption is, moreover, supported by the low amylose content (≤20%), which gives to mango almond starch a low susceptibility to retrogradation. Proteins and lipids, with respective content ranges of 4-5% and 7-12%, are likely to contribute to the hypothetical technological functionality in breadmaking, and at the same time, they contribute to nutritional value of mango almonds.

Analysis of the oil composition shows a fatty acid profile that is practically at parity between unsaturated and saturated fatty acids, ranged, respectively, between 48%–59% and 40%–52%, mainly carried by palmitic (10–17%), stearic (23–40%), oleic (39–46%), and linoleic (1–10%) acids, depending on mango varieties. The high content of saturated fatty acids in all varieties of almonds (40-52%) suggests a potential for reducing bread staling, as these fatty acids are likely to form a complex with amylose, limiting thus the retrogradation of the polysaccharide, the main carrier of the staling phenomenon [23].

Amylase activity is an important parameter in reducing the rate of bread stale [24]. Almonds of all mango varieties displayed amylase activity, with Haden and Brook's varieties displaying the highest activity, while Springfield variety showed the lowest. Considering the analytical conditions of the test, the amylolytic activity, detected at 60°C, could be considered to be carried primarily by *α*-amylase.

### 3.2. Functional Properties of Mango Almond Flours

From functional point of view, mango almond flours are characterized by a relatively high WAC and OAC (Table 2), corresponding to approximately double or even triple of the values observed in wheat. Indeed, the WAC values vary in the ranges of 175%-211% and 236%-315%, respectively, for native and delipidated mango almond flours, against 140% for wheat flour [25], findings compatible with the high amylopectin content of mango almond starch. OAC values are in the ranges of 125%-143% and 185%-254%, respectively, for native and delipidated mango almond flours versus 90% for wheat flour [26]. However, the WOAI (water oil absorption index), calculated as the WAC/OAC ratio [27], gives comparable values, between 1.3 and 1.5, for all almond flours (native and delipidated). This index, generally considered as an indicator of emulsifying capacity, is relatively low and compatible with the low emulsifying activity (EA) of mango almond flours. Furthermore, the weak emulsion observed is not stable, particularly when the flour is delipidated. These findings show that the significant absorption of water and oil by mango almond flours is not indicative of a functional synergy of the flour constituents. It should, however, be noted that the correlation between WOAI and the emulsifying capacity is valid especially when the water absorption is carried by proteins, which is not the case in mango almond flours, where starch is the carrier of water absorption. In fact, the large increase in WAC (doubling) and OAC in delipidated mango kernel flours points to a possible surface interaction between starch and mango almond lipids. It is in fact recognized that amylose-lipid complexes can form naturally in the starch granule through weak energy surface binding, due to the fact that the helical structure of the amylose chain is characterized by an orientation of the hydrophilic groups towards the outer surface of the starch helix, while the internal cavity forms a tube with a hydrophobic tendency in which the lipid molecules are easily lodged [28] and are easily removed by hexane treatment. It can be assumed for this purpose that during defatting, the helical chain cavities of amylose are freed from the oil that had naturally accumulated there. As a result, in a water-saturated medium, the hydrophilic groups are much more available for water (strong increase in WAC), and in an oil-saturated medium, the hydrophobic cavities of the amylose helices are more available to accumulate lipid molecules (hence increase in OAC).

Overall, the chemical compositions of mangoes, in particular their high starch content, their richness in amylopectin, the possibility of the formation of starch-lipid complexes, and the significant presence of phenolic compounds, are all elements that militate in favor of a potential impact of these flours on the rheology of the dough during breadmaking.

### 3.3. Technofunctional Properties of Wheat and Mango Almond Flour Blends

#### 3.3.1. Hydrothermal Behavior of Individual Mango Almond Flours and Starches in Dispersed Aqueous Solution (RVA Measurements)

**Figure 1:**
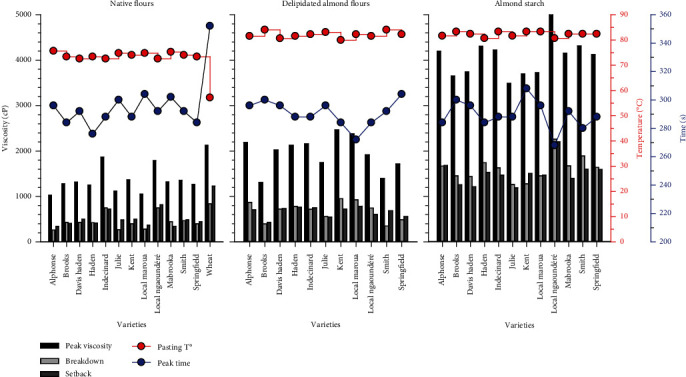
Pasting indicators of mango almonds flours (native and delipidated) and starch from different mango varieties and wheat flour.

The RVA indicators of native and delipidated flours, as well as of starch from mango almond flours, are shown in Figure 1. The profile of wheat flour is included for comparison with native mango almond flours. During hydrothermal treatment under shear, all mango almond flours (native and delipidated), as well as their starches, start gelatinization (Pasting Temperature) above 80°C, a value greater than the pasting temperature of wheat flour which is around 63°C. In addition, native mango almond flours (Figure 1(a)) reach their peak viscosity more quickly (260 s ≤ Peak time ≤ 270 s) than wheat flour (320 s). In this regard, the flours of Haden and Local Ngaoundéré varieties exhibit the highest swelling comparable to that of wheat flour. This contrasting rheological behavior, also shown on the viscogram of starches extracted from mango almond flours (Figure 1(c)), may be attributed at first glance to varietal diversity of mangoes reflected above by the variability of their chemical composition and the structure of their starch.

The defatting of almond flour results in an increasing trend of “Peak Time” and “Peak Viscosity” which are approaching the values of wheat flour (Figure 1(b)). On the other hand, the impact of defatting on breakdown and setback remains relatively contrasted, insofar as these indicators are relatively higher for certain delipidated mango almond flours (Alphonse, Davis Haden, Haden, Kent, Local Maroua) and stable for others (Brooks, Indécinard, Julie, Local Ngaoundéré, and Springfield). These results seem to indicate a contrasting starch-lipid interaction in the different mango almond flours. It should be noted that the pasting temperature remains constant for all almond flours, regardless of the treatment (native and delipidated), and corresponds to the pasting temperature of the flour starches, which indicates that the hydrothermal properties of mango almond flours in a dispersed medium are carried exclusively by their starch.

If the relative variability of the rheological indicators is certainly linked to the structural variability of the starch in the samples, in particular the amylose/amylopectin ratio, which is likely to justify the variability of their water absorption capacity (Table 2), it could also be associated with the presence of nonpolysaccharide material, in particular, lipids and proteins which interact with starch molecules through adsorption at the surface layer of starch granule (starch surface protein and lipid complexes), entrapment within starch granules or complexation with amylose (starch interior protein and lipid complexes) [9], and result in a significant impact on starch properties and applications. The pasting behavior of mango almond flours and starches (Figure 1) seems to reflect these interactions.

In the native mango almond flours, the relatively low pasting indicators (peak viscosity, breakdown, and setback) could be attributed to constraints imposed by nonpolysaccharide materials which limit the swelling of granules (Figure 1(a)). Defatting of flours using hexane removes only starch surface complexes, which justifies low increase of pasting indicators (Figure 1(b)), and also the high WAC and OAC observed previously (Table 2). But the use of sodium dodecyl sulfate (SDS) for purification of starch during extraction [10] is a disruptive method which removes starch interior proteins and lipids, contributing to increase the granule expansion (Figure 1(c)) in such an extensive way that the resultant swollen granules are relatively fragile and generate higher breakdown and setback. Hu et al. [9] showed that molecular structure of starch is not affected by the disruptive effect of SDS, which may explain the fact that amylose released from complexation with nonpolysaccharide materials readily gelatinizes and retrogrades.

#### 3.3.2. Hydrothermal Behavior of Mango Almonds and Wheat Flour Blends

**Figure 2:**
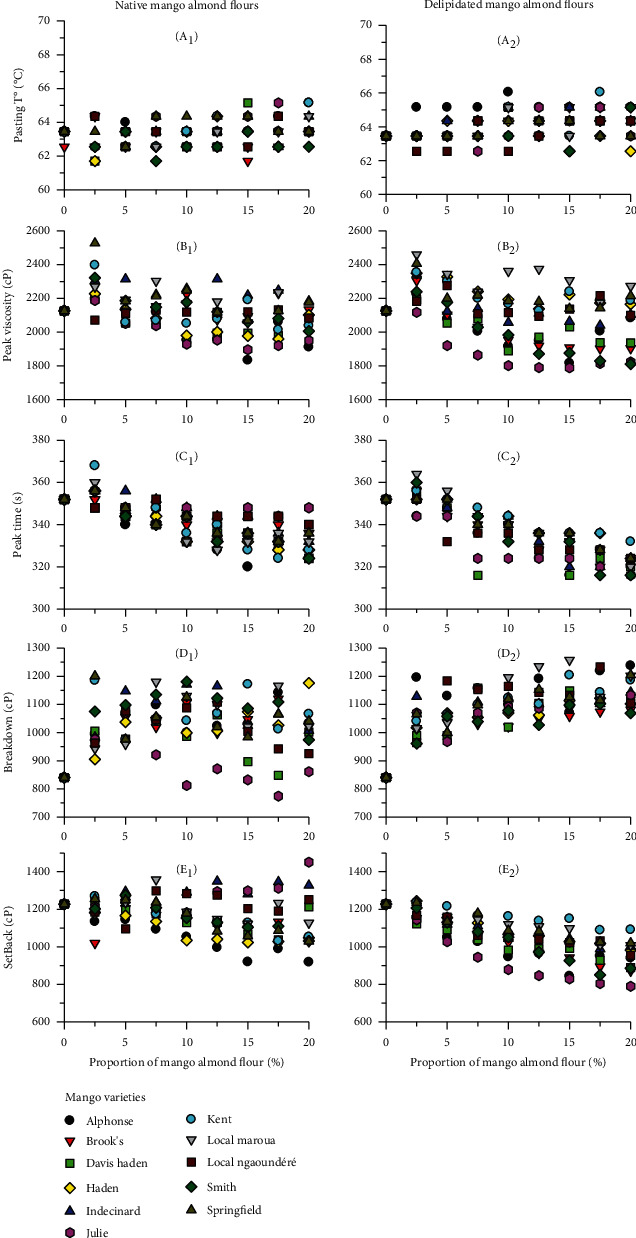
Pasting behavior of wheat flour partially substituted with mango almond flours from different mango varieties.

Partial substitution of wheat by mango almond flours (0-20%) does not induce a significant modification of the pasting temperature which, for all substitution levels, is around 63-65°C (Figure 2(a)_1&2_). On the other hand, the maximum swelling (peak viscosity) seems subject to a substitution threshold effect, with a tendency of swelling increase for substitution between 0 and 2.5%, followed by a gradual decrease beyond that substitution level, to apparently stabilize for substitutions between 10% and 15%, values beyond which no significant variation in swelling is observed (Figure 2(b)_1&2_). The same is true for the gelatinization time (Figure 2(c)_1&2_). This behavior of composite flours during gelatinization could be the result of a competition of the compounds present for water, following the observations of Collar et al. [29], who showed that the partial substitution of wheat flour by fiber components with a high-water absorption capacity reduces the amplitude and the gelatinization kinetics. In this regard, mango almond flours, which require more water than wheat, would lower the gelatinization. The profiles obtained during this gelatinization phase are almost identical between the substitutions by native and delipidated mango almond flours.

Breakdown of starch granules after swelling has a higher amplitude compared to wheat for all substitutions (Figure 2(d)_1&2_), which would indicate low heat resistance of the starch granules in composite flours. This behavior presents an apparent threshold, in terms of substitution rate, around 2.5% and 5% substitution, above which an inversion (or stabilization) of the breakdown evolution is observed, depending on the mango variety. However, there is a relative difference between wheat flours substituted by native almond flours and those substituted by delipidated flours. The increase in the amplitude of granule bursting is clearer and more regular in the blends containing the delipidated flours (Figure 2(d)_2_), the evolution being linear as a function of the degree of substitution, with all samples presenting a comparable pace. On the other hand, the amplitude of the breakdown is contrasted in blends containing native flours, for which beyond the apparent threshold, the magnitude of granule bursting remains relatively stable for some varieties or decreases for others, sometimes to the level or even below the bursting level of the gelatinized wheat starch granule (Figure 2(d)_1_). The latter is for instance, the case of the blend with native almond flour of the Julie variety. This flour is also the one which presented, in individual hydrothermal treatment, the lowest breakdown (Figure 1). Bringing together the findings on breakdown variation and on the reduction tendency of peak viscosity of the blends could lead to suppose that in the mixtures, it is the starch of the mango almond flours which would have first reached their maximum swelling (due to their higher hygroscopicity) and would have burst. This seems normal since mango almond flours have a shorter swelling time than wheat.

The contrast between native and delipidated mango almond flours is also observed on the setback behavior of the flour blends, comparable to what is observed above for the breakdown. If the retrogradation of all blends containing delipidated flours decreases regularly with the increase in the substitution rate, a double trend is noted with native mango almond flours; the substitution of wheat by native flours of Julie, Indécinard, and local varieties (Maroua and Ngaoundéré) increases the retrogradation (Figure 2(e)_1_) while all the flours of the other varieties reduce it.

The above findings suggest that the functional use of mango almond flours would be variety dependent and should take into account, in addition to the rheological properties of the flours, their pretreatment conditions (defatting or not), and their chemical composition and potential interactions between the constituents, depending on the envisaged processes. It should be noted that these functional conditions of mango almond flours, provided by the RVA studies, respond to applications in dispersed medium and could well be perceived differently in an application such as breadmaking where the dough is less fluid.

#### 3.3.3. Contribution of Mango Almond Flours in the Rheology of Bread Dough (Alveographic Measurements)

**Figure 3:**
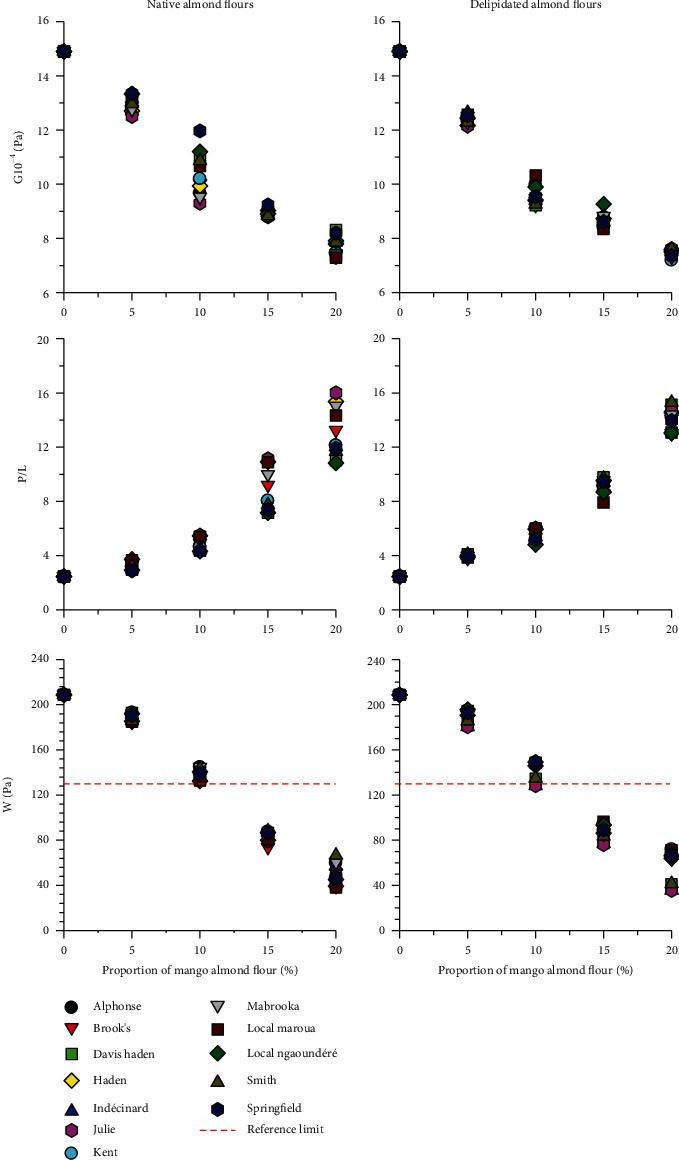
Alveographic behavior of wheat flour partially substituted by mango almond flours from different mango varieties.

The partial substitution of wheat by mango almond flours results in an overall increase in the tenacity (P) of the dough which shifts the tenacity-extensibility balance (P/L) in favor of tenacity. This results in a decrease of the swelling index (G) and of the baking strength (W) (Figure 3). These variations are intensified with increasing substitution rate and can range up to about 45%, 80%, and 60%, respectively, of the swelling, toughness, and baking strength of 100% wheat flour. In addition, the variety of mango and the native or delipidated state of the almond flour do not seem to induce a highly significant effect on the alveographic profiles.

On a hypothetical basis, the low expansion induced by the substituted flours could be attributed to the high-water absorption capacity of the mango almond flours, which suggests that, under the conditions of the analysis, wheat gluten, due to competition for water with mango almond flour, would not have had the possibility to absorb enough water for its swelling, like what was observed above in the RVA monitoring of the hydrothermal behavior of substituted flours (Figure 2). A probable consequence of this would be a weak cohesion between the particles of wheat flour and mango almonds. If this assumption is true, it means that, in order to use the substituted flours in breadmaking, it would appear useful to manage differently the quantity of water to be used for substituted flours and eventually the mixing factors. Beyond this hypothesis, the phenolic compounds of the mango almond can also appear as a factor of resistance to the extension of the composite paste, insofar as they are likely to interact with the gluten network and strengthen it [30].

Comparative analysis of the behavior of our composite flours with respect to the technological standards applicable in different countries shows that:

- All our flours, including the reference wheat used, have a swelling index (G) and a tenacity-extensibility ratio (P/L) outside the current standards (20 *cm*^3^ ≤ *G* ≤ 24 *cm*^3^. 0.45 ≤ *P*/*L* ≤ 0.7) from countries such as France and Algeria [31]. The noncompliance of the wheat flour used by local bakers, and taken in this study as control, is probably due to the fact that these flours often come from a mixture of different wheat varieties
- However, up to 10% wheat substitution, wheat-mango almond composite flours have a strength within the range authorized in various countries (*W* ≥ 130.10^−4^ *J*) [31, 32]. It can therefore be assumed hypothetically that a substitution up to 10% of wheat by mango almond flour is likely to be used suitably in breadmaking

**Figure 4:**
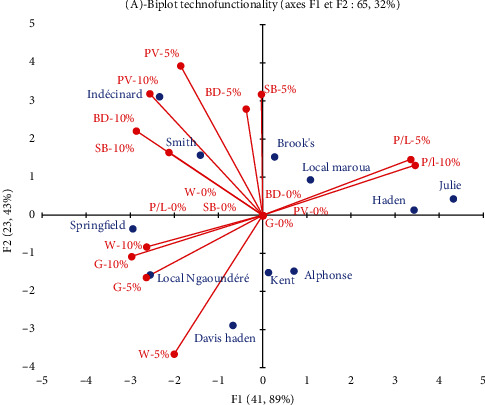
Variability of pasting and viscoelastic properties of almond flours from different mango varieties at different levels of substitution (0–10%) in wheat flour.

The integrated comparison of the technofunctional behavior (RVA and Alveogram) of wheat-mango almond flour blends, in the substitution range between 0% and 10%, allows (Figure 4) to divide the different mango varieties into 3 groups in relation to the functionality of their almond flours:

- The almonds of the Indécinard and Smith varieties contribute mainly to the hydrothermal swelling (RVA) of the mixtures, with the highest values of PV, BD, and SB, regardless of the substitution rate. The retrogradation induced by these flours appears as a negative indicator in dough rheology for breadmaking, insofar as a high susceptibility to retrogradation is likely, in breadmaking, to lead to the rapid stale of the bread [33]
- The almonds of the Springfield Local Ngaoundéré and Davis Haden varieties contribute to the swelling (G) and to the strength (W) of the dough, which is an indicator of an acceptable rise of the dough during fermentation
- The almonds of the Brook's, Local Maroua, Julie, Haden and, to a lesser extent, Kent, and Alphonse varieties are characterized by their impact on the increase of the tenacity-extensibility ratio (P/L) of the bread dough, which may lead to a deleterious behavior of the bread dough

Since the technofunctionality is carried by the composition of the mango almond flours, the integrated comparison of the latter in relation to the constituents liable to influence the functionality of the flours shows (Table 1) that, although all the Mango almonds contain, at varying degrees, the functional constituents for breadmaking, the varieties of the second and third groups defined above (Figure 4) are among the richest in compounds of technological interest in breadmaking.

## 4. Conclusion

The chemical compositions of mango almonds (in particular their high starch content, richness in amylopectin, quality and quantity of lipid, and significant presence of phenolic compounds), coupled to their technofunctional (pasting and viscoelastic) properties, militate in favor of the use of their flour as partial substitute of wheat, at a maximum level of 10%, in breadmaking, with respect to potential reduction of bread staling. Nine varieties of mango from savanna regions of Central Africa appear suitable in this regard. Meanwhile, based on the varietal and property diversity of mango, breadmaking processing factors may need to be optimized, in order to define the best varieties, substitution and processing conditions for convenient composite bread to be able to resist the staling phenomenon.

## Figures and Tables

**Table 1:** Chemical composition of mango almonds.

Mango varieties	Components (g/100 g DW)								
	Proteins	Lipids	Free sugars	Starch	Amylose	Fibers	Ash	Total phenolic compounds	Tannins
Alphonse	5.63 ± 0.33^bc^	8.86 ± 0.22^b^	3.57 ± 0.06^ab^	63.29 ± 0.54^def^	11.90 ± 0.15^b^	0.56 ± 0.26^a^	2.17 ± 0.38^bc^	4.56 ± 0.59^c^	3.17 ± 0.60^d^
Brook's	6.40 ± 0.32^d^	9.42 ± 0.21^bc^	3.32 ± 0.04^ab^	64.49 ± 0.44^fg^	11.87 ± 0.46^b^	1.88 ± 0.32^c^	3.39 ± 0.23^e^	4.51 ± 0.07^b^	1.46 ± 0.86^ab^
Davis Haden	5.21 ± 0.27^ab^	13.06 ± 0.81^e^	7.04 ± 0.90^g^	61.01 ± 0.54^abc^	9.42 ± 0.37^a^	0.56 ± 0.26^a^	2.14 ± 0.40^abc^	2.12 ± 0.10^a^	0.69 ± 0.13^a^
Haden	6.11 ± 0.14^cd^	9.43 ± 0.30^bc^	3.28 ± 0.02^a^	62.85 ± 0.83^def^	15.88 ± 0.24^f^	1.53 ± 0.53^bc^	2.33 ± 0.21^bcd^	3.61 ± 0.01^bc^	2.26 ± 0.05^bcd^
Indécinard	9.05 ± 0.29^f^	9.10 ± 0.47^b^	3.10 ± 0.22^a^	60.28 ± 1.53^ab^	13.41 ± 0.11^c^	1.49 ± 0.52^bc^	1.53 ± 0.16^a^	3.79 ± 0.62^bc^	2.44 ± 0.67^bcd^
Julie	6.39 ± 0.27^d^	9.79 ± 0.16^bcd^	4.59 ± 0.53^cde^	65.81 ± 0.96^g^	14.46 ± 0.21^e^	2.96 ± 0.12^d^	3.44 ± 0.12^ef^	4.35 ± 0.10^bc^	2.83 ± 0.14^cd^
Kent	6.45 ± 0.35^de^	12.08 ± 0.26^e^	4.91 ± 0.14^def^	59.35 ± 0.83^a^	13.80 ± 0.28^cd^	1.31 ± 0.26^abc^	4.06 ± 0.38^f^	1.54 ± 0.43^a^	0.31 ± 0.02^a^
Local Maroua	4.88 ± 0.29^a^	12.79 ± 0.88^e^	4.22 ± 0.44^bcd^	62.64 ± 2.16^cde^	14.17 ± 0.37^de^	2.61 ± 0.52^d^	1.77 ± 0.19^ab^	5.46 ± 0.20^d^	4.04 ± 0.20^d^
Local Ngaoundéré	5.43 ± 0.29^ab^	10.45 ± 0.27^cd^	3.98 ± 0.20^abc^	63.78 ± 0.35^ef^	17.73 ± 0.11^g^	1.48 ± 0.29^bc^	2.86 ± 0.38^de^	7.44 ± 0.46^e^	5.91 ± 0.46^e^
Mabrooka	7.02 ± 0.14	7.45 ± 0.74^a^	5.17 ± 0.17^ef^	63.46 ± 0.60^ef^	15.89 ± 0.11^f^	0.94 ± 0.26^ab^	2.91 ± 0.25^de^	3.56 ± 0.13^b^	2.03 ± 0.21^bc^
Smith	5.61 ± 0.22^bc^	9.77 ± 1.21^bcd^	5.59 ± 0.24^f^	62.77 ± 1.48^cdef^	15.59 ± 0.23^f^	1.11 ± 0.43^abc^	2.89 ± 0.24^de^	5.70 ± 0.13^d^	4.33 ± 0.12^d^
Springfield	5.71 ± 0.13^bc^	10.66 ± 0.36^d^	3.60 ± 0.12^ab^	61.64 ± 0.88^bcd^	15.96 ± 0.48^f^	0.93 ± 0.26^ab^	2.59 ± 0.20^cd^	3.93 ± 0.13^bc^	2.46 ± 0.10^bcd^

(a–c) In the same column, values with the same letter in superscript are not significantly different (*p* = 0.05).

**Table 2:** Some functional properties of mango almond flours.

Varieties	WAC (%)		OAC (%)		WOAI		EA (%)		ES (%)	
	Native flour	Delipidated flour	Native flour	Delipidated flour	Native flour	Delipidated flour	Native flour	Delipidated flour	Native flour	Delipidated flour
Alphonse	197.07 ± 8.38^cd^	307.92 ± 7.50^de^	129.48 ± 4.38^ab^	185.27 ± 3.02^a^	1.52	1.66	2.55 ± 0.36^b^	1.53 ± 0.25^e^	1.23 ± 0.35^ab^	0
Brook's	221.26 ± 7.70^f^	284.95 ± 5.12^c^	130.50 ± 1.70^abc^	187.58 ± 4.37^ab^	1.70	1.52	1.5 ± 0.1^a^	0.62 ± 0.12^a^	0.43 ± 0.15^a^	0
Davis Haden	186.57 ± 5.42^abc^	343.39 ± 6.42^f^	139.85 ± 2.41^de^	254.85 ± 4.96^h^	1.33	1.35	1.73 ± 0.21^a^	1.10 ± 0.10^c^	0.43 ± 0.21^a^	0
Haden	194.37 ± 9.97^bcd^	311.85 ± 9.20^de^	126.09 ± 4.83^a^	208.05 ± 9.05^ef^	1.54	1.50	10.36 ± 0.73^d^	1.07 ± 0.15^bc^	3.13 ± 0.87^c^	0
Indécinard	182.15 ± 6.23^ab^	282.61 ± 6.71^c^	127.62 ± 2.98^a^	193.99 ± 4.84^bc^	1.43	1.46	4.05 ± 0.16^c^	1.25 ± 0.20^cd^	1.67 ± 0.31^b^	0
Julie	236.55 ± 8.80^g^	255.84 ± 5.34^b^	125.84 ± 4.46^a^	206.07 ± 4.96^de^	1.88	1.24	3.77 ± 0.25^c^	0.74 ± 0.06^a^	1.43 ± 0.51^b^	0
Kent	175.31 ± 5.31^a^	263.99 ± 5.59^b^	126.21 ± 3.79^a^	235.18 ± 4.70^g^	1.39	1.12	2.00 ± 0.21^ab^	1.27 ± 0.25^cd^	0.57 ± 0.16^a^	0
Local Maroua	196.19 ± 10.24^cd^	258.53 ± 7.40^b^	128.52 ± 4.66^ab^	197.60 ± 2.37^cd^	1.53	1.31	14.70 ± 1.03^e^	1.44 ± 0.06^de^	5.37 ± 0.97^d^	0
Local Ngaoundéré	211.29 ± 10.24^ef^	304.34 ± 5.97^d^	137.16 ± 2.01^cde^	233.19 ± 6.13^g^	1.54	1.31	10.45 ± 0.77^d^	1.20 ± 0.16^cd^	4.63 ± 0.61^d^	0
Mabrooka	203.52 ± 8.35^de^	236.41 ± 5.11^a^	135.56 ± 7.11^bcd^	205.33 ± 5.31^de^	1.50	1.15	3.94 ± 0.12^c^	1.24 ± 0.07^cd^	1.63 ± 0.26^b^	0
Smith	192.10 ± 3.57^bcd^	289.12 ± 3.18^c^	126.71 ± 4.00^a^	215.12 ± 1.64^f^	1.52	1.34	2.23 ± 0.20^ab^	0.82 ± 0.12^ab^	0.95 ± 0.23^ab^	0
Springfield	188.89 ± 8.84^bc^	315.83 ± 8.92^e^	143.44 ± 7.17^e^	254.91 ± 5.10^h^	1.32	1.24	3.78 ± 0.11^c^	1.20 ± 0.13^cd^	1.53 ± 0.25^b^	0

(a–c) In the same column, values with the same letter in superscript are not significantly different (*p* = 0.05). WAC: water absorption capacity; OAC: oil absorption capacity; EA: emulsifying activity; ES: emulsion stability.

## Data Availability

The data used to support the results of this study are available from the corresponding author upon request.
